# Novelties in *Begonia* sect. *Coelocentrum*: *B. longgangensis* and *B. ferox* from limestone areas in Guangxi, China

**DOI:** 10.1186/1999-3110-54-44

**Published:** 2013-10-07

**Authors:** Ching-I Peng, Hsun-An Yang, Yoshiko Kono, Kuo-Fang Chung, Yu-Song Huang, Wang-Hui Wu, Yan Liu

**Affiliations:** 1 Herbarium (HAST), Biodiversity Research Center, Academia Sinica, Taipei 115, Nangang, Taiwan; 2 School of Forestry and Resource Conservation, National Taiwan University, Taipei 106, Taiwan; 3 Guangxi Institute of Botany, Guangxi Zhuangzu Autonomous Region and the Chinese Academy of Sciences, Guilin, 541006 China

**Keywords:** *Begonia longgangensis*, *Begonia ferox*, Begoniaceae, China, Chromosome number, Limestone, Sect. *Coelocentrum*, Triploid

## Abstract

**Background:**

The spectacular karst limestone landscape in Guangxi harbors high-level diversity and endemism of *Begonia* species, especially those of sect. *Coelocentrum*. In continuation of our studies in this area, we report the discovery of two attractive new species from southwestern Guangxi: *Begonia longgangensis* and *B. ferox.*

**Results:**

*Begonia longgangensis* resembles *B. liuyanii*, also from Longgang Nature Reserve, in the broadly ovate to suborbicular leaf blade, differing by the much smaller leaves, subglabrous leaf surface, pink flowers, dichasial cymes and the remarkably long stolons sent out from rhizomes. Unexpectedly, both diploid (2*n* = 30) and triploid counts (2*n* = 45) were observed in plants collected from the type locality. *Begonia ferox* probably has the most prominent bullate leaves for the genus. In this aspect, it is similar to *B. nahangensis* reported from northern Vietnam recently, but is readily distinguishable by the ovate, chartaceous leaves with an acuminate apex; tomentose peduncle not exceeding petioles; and the much larger stature in vegetative parts. A diploid count of 2*n* = 30 was determined for this unique new species.

**Conclusions:**

All available data support the recognition of the two new species. *Begonia longgangensis* has remarkably long stolons and *B. ferox* is characterized by the prominent bullate leaves. Line drawings, color plates and comparisons are provided to aid in identification of the novelties.

**Electronic supplementary material:**

The online version of this article (doi:10.1186/1999-3110-54-44) contains supplementary material, which is available to authorized users.

## Background

The area from South China to North Vietnam harbors very high levels of biological diversity (Sodhi et al., 2004). Inventorying the biological diversity in this region, however, is largely insufficient (Hou et al., 2010). Numerous new taxa have been described from there in recent years, especially from the limestone areas, e.g. *Aspidistra*: Hou et al., 2009, Lin et al., 2010, Liu et al., 2011, 2013; *Begonia*: Fang et al., 2006, Ku et al., 2006, 2008, Liu et al., 2005, 2007, Peng et al., 2005a, b, 2006, 2007, 2008a, b, 2010, 2012; *Primulina*: Xu et al., 2012, 2013; *Oreocharis*: Liu et al., 2012; *Polystichum*: He and Zhang 2011, Zhang and He, 2009a, b, Zhang et al., 2010. In continuation of our studies of Chinese *Begonia*, we report the discovery of two additional new species, *B. longgangensis* and *B. ferox*, from limestone karsts in southwestern Guangxi.

## Methods

### Chromosome preparations

Somatic chromosomes of the new species, *Begonia longgangensis* (*Peng et al.*, *22930*) and *B. ferox* (*Peng et al., 22956*), were examined using root tips. The methods of pretreatment, fixation and staining for chromosome observations followed Hughes et al. (2011). Classification of the chromosome complements based on centromere position at mitotic metaphase follows Levan et al. (1964). Voucher specimens have been deposited in Herbarium, Biodiversity Research Center, Academia Sinica, Taipei (HAST).

## Results and discussion

### Species description

**Figure 1:**
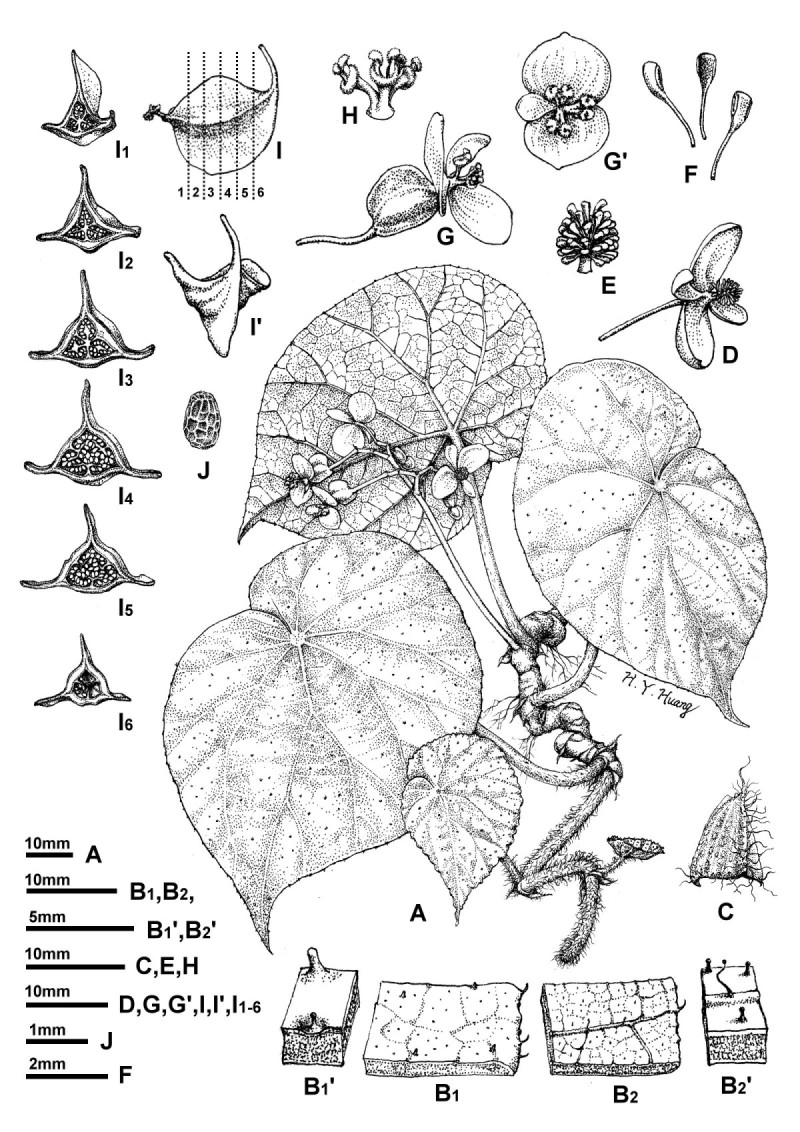
***Begonia longgangensis*** **C.-I Peng & Yan Liu. A**, Habit; **B1**, **B1’**, Leaf adaxial surface, showing margin and indumentum, **B2**, **B2’**, Leaf abaxial surface; **C**, Stipule; **D**, Staminate flower; **E**, Androecium; **F**, Stamens; **G**, Carpellate flower, side view, **G’**, face view; **H**, Style and stigmas; **I**, **I’**, Fruit; **I**, **I1-6**, Serial cross sections of a developing fruit; **J**, Seed. All from *C.-I Peng et al. 22930* (HAST).

**Figure 2:**
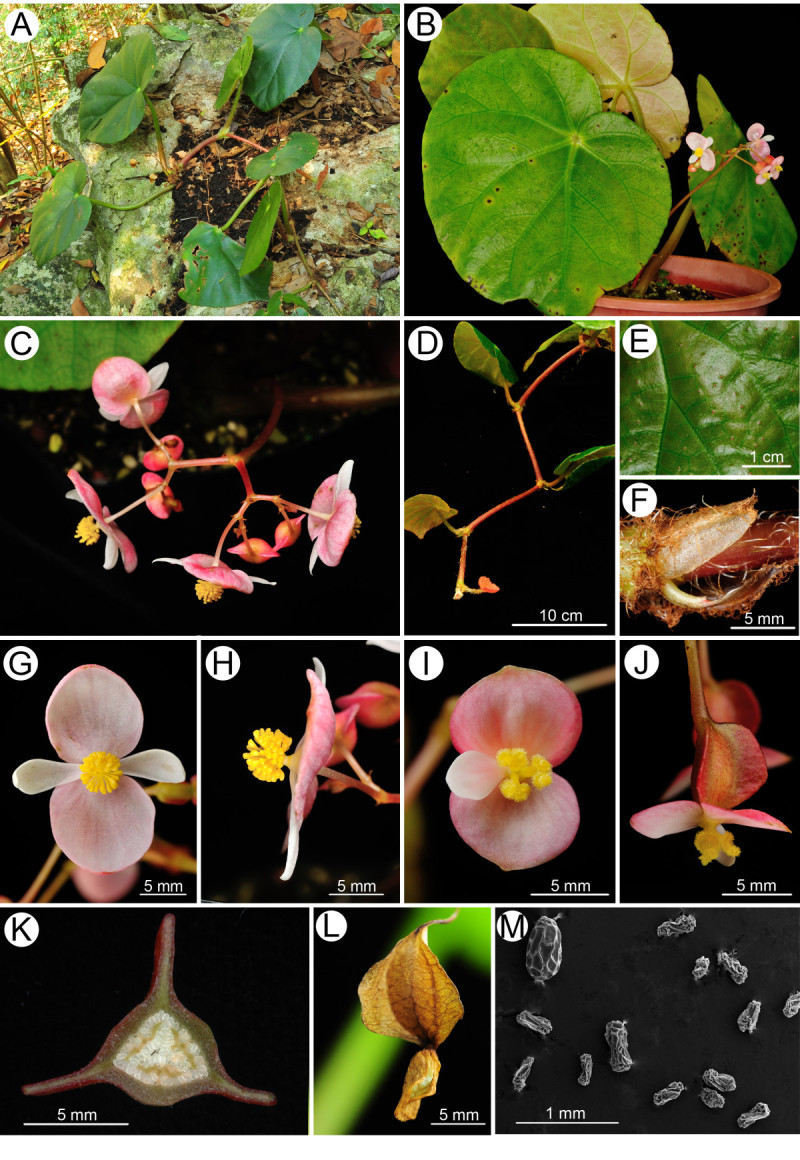
***Begonia longgangensis*** **C.-I Peng & Yan Liu. A**, Habit and habitat; **B**, Cultivated plant at anthesis; **C**, Inflorescence; **D**, Stolon; **E**, Leaf adaxial surface; **F**, Stipule; **G**, Staminate flower, face view; **H**, Staminate flower, side view; **I**, Carpellate flower, face view; **J**, Carpellate flower, side view; **K**, Middle cross section of ovary; **L**, Capsule with persistent tepals; **M**, Seed SEM microphotograph (abortive seeds from a field-collected capsule). All from *C.-I Peng et al. 22930* (HAST).

**1.** ***Begonia longgangensis*** C.-I Peng & Yan Liu, sp. nov. (sect. *Coelocentrum*) —TYPE: CHINA, Guangxi Zhuangzu Autonomous Region, Longgang Nature Reserve, elev. ca. 170 m, on limestone hill and cliff, 18 April 2011, *Ching-I Peng, Kuo-Fang Chung, Yu-Song Huang, Bo Pan & Sheng-Yuan Liu 22930* (holotype: HAST; isotype: IBK).  Figures 1 and 2.

**Figure 3:**
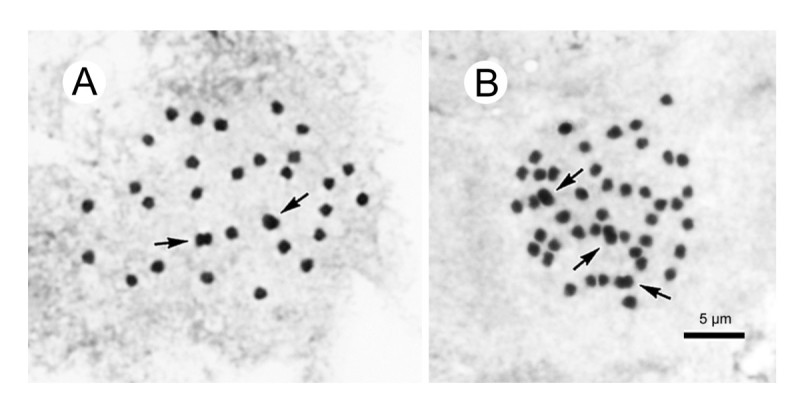
**Somatic chromosomes at metaphase of** ***Begonia longgangensis*** **(from Peng et al. 22930, HAST). A** and **B**, micrographs: **A**, 2*n* = 30; **B**, 2*n* = 45. Arrows indicate longer metacentric chromosomes.

Monoecious herb, rhizomatous initially and becoming stoloniferous. *Rhizomes* 1–2 cm thick, internodes 1–1.5 cm long, sending out stolons to 150 cm or longer, 0.5-0.7 cm thick, villous, internodes 8-13(-17) cm long. *Stipules* eventually deciduous, ovate-triangular, 1–1.8 cm long, 1–1.2 cm wide, margin entire, strongly keeled, abaxially hairy along midrib, apex aristate, arista 0.4-0.7 cm long. *Leaves* alternate, petioles terete, (6-)9-18(-25) cm long, 0.7 cm thick, green or reddish, white villous when young, becoming brown tomentose, glabrescent; leaf blade asymmetric, broadly ovate to suborbicular, (10-)14.5-20 cm long, (7-)10.5-15 wide, apex acuminate, base strongly obliquely cordate, margin initially serrulate and shortly ciliate, becoming repand when mature, subcoriaceous, adaxially green, sparsely reddish-scabridulous and with minute processes, abaxially pale, tomentose on veins. *Inflorescences* axillary, arising directly from rhizome or stolons, dichasial cymes branched 3–4 times, peduncle 9–14 cm long, glabrous; bracts caducous. *Staminate flower*: pedicel 1.2-1.5 cm long, tepals 4, outer 2 broadly ovate, pinkish, 1–1.4 cm long, 1.2-1.4 cm wide, inner 2 elliptic, white, 0.8-1.2 cm long, 0.4-0.5 cm wide; androecium actinomorphic, spherical, ca. 0.5 cm across; stamens 40–60; filaments fused into a column ca. 0.2 cm long; anthers 2-locular, obovate, connective obtuse at apex. *Carpellate flower*: pedicel 1.4-1.9 cm long, tepals 3, outer 2 broadly ovate to suborbicular, pinkish, 0.7-1.2 cm long, 0.8-1.2 cm wide, inner 1 elliptic, white, 0.6-1 cm long, 0.3-0.5 cm wide; ovary trigonous-ellipsoid, 0.7-1.1 cm long, 0.3-0.5 cm across (wings excluded), reddish, glabrous, 3-winged; wings unequal, greenish, lateral wings narrower, 0.2-0.3 cm tall, abaxial wing crescent-shaped, 0.3-0.5 cm tall; styles 3, fused ca. 0.5 cm long at base, stigma spirally twisted. *Capsules* nodding, 0.9-1.3 cm long, 0.5 cm across (wings excluded), apex with persistent tepals; wings unequal, lateral wings 0.3 cm tall, abaxial wing crescent-shaped, 0.3-0.6 cm tall; seed plump or abortive. Somatic chromosome number, 2*n* = 30, 45 (Figure 3).

#### Chromosome cytology

Our study of the somatic chromosomes at metaphase of *Begonia longgangensis* revealed different ploidy levels in plants of the same population (2*n* = 30, 45) (Figure 3). Previously, a few cases were documented in the genus *Begonia* in which infraspecific polyploidy occurs, e.g. *B. monophylla* with 2*n* = 28, 56 (Legro and Doorenbos, 1969); *B. rex* with 2*n* = 24, 48 (Sharma, 1970); *B. squamulosa* with 2*n* = 38, 76 (Arends, 1992). This is the first confirmed report of a naturally occurring triploid in *Begonia* sect. *Coelocentrum*.

The diploid (2*n* = 30) has two longer metacentric chromosomes ca. 1.4-1.5 μm long (Figure 3A: arrows) and 28 shorter chromosomes ca. 1.0-1.2 μm long. By contrast, the triploid (2*n* = 45) has three longer metacentric chromosomes, ca. 1.7 μm long (Figure 3B: arrows), and 42 shorter chromosomes, ca. 0.9-1.3 μm long. The centromere positions of most chromosomes in both diploid and triploid plants could not be determined. Satellites were not observed.

All 19 species of *Begonia* sect. *Coelocentrum* that we studied cytologically uniformly had the chromosome number of 2*n* = 30 (Peng et al., 2012), of which seven were known to have bimodal karyotypes with two longer metacentric chromosomes in the chromosome complement. Since there are diploid and triploid plants within a natural population of *B. longgangensis*, by comparison of the number of longer metacentric chromosomes, the 2*n* = 45 plant likely represents intraspecific autotriploid.

#### Additional specimens examined

CHINA. Guangxi Zhuangzu Autonomous Region, Longzhou County, Zhubu Xiang, Longgang Cun, Lenglei Tun, 6 Oct 1979, *Longgang Exped. 20386A* (GXMI); Longzhou County, Longgang Nature Reserve, elev. ca. 170 m, on limestone hill and cliff, 18 April 2011, flowering specimen pressed from cultivated plant in 28 May 2013, *Ching-I Peng, Kuo-Fang Chung, Yu-Song Huang, Bo Pan & Sheng-Yuan Liu 22930-A* (HAST).

#### Ecology and distribution

**Figure 4:**
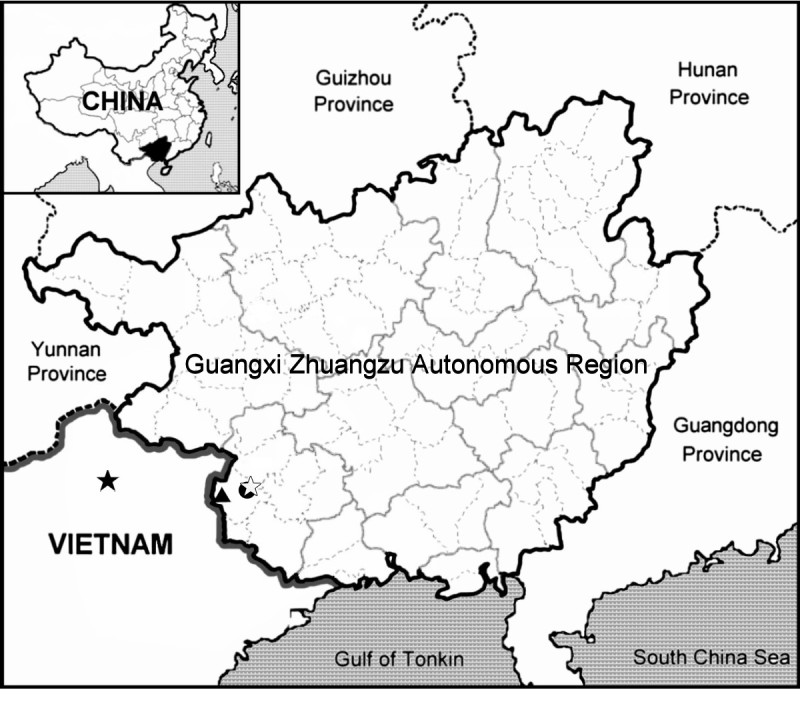
**Distribution of** ***Begonia nonggangensis*** **(●),** ***B. liuyanii*** **(☆),** ***B. ferox*** **(▲) in Guangxi Zhuangzu Autonomous Region, China and** ***B. nahangensis*** **(★) in Vietnam.**

Stoloniferous herb on jagged limestone rocks in evergreen broadleaf forest; known only from the type locality in Longzhou County, Longgang Nature Reserve, Guangxi, China (Figure 4).

#### Phenology

Flowering from March to June; fruiting from May to August.

#### Etymology

The specific epithet is derived from the type locality, Longgang Nature Reserve, Guangxi.

#### Notes

*Begonia longgangensis* somewhat resembles *B. liuyanii* in the broadly ovate to suborbicular leaf shape (Peng et al., 2005), differing in the rhizomes that send out elongate stolons; much smaller leaves, sparsely reddish-scabridulous leaf adaxial surface with minute processes, tomentose only on veins of leaf abaxial surface; dichasial inflorescence, pink flowers and glabrous peduncle, tepal and ovaries. A detailed comparison is provided in Table 1.

**Table 1:** **Comparison of** ***Begonia longgangensis*** **with** ***B. liuyanii***

	***Begonia longgangensis***(Figures1 and2)	***Begonia liuyanii***(Peng ***et al.***, 2005: Figures1 and2)
Rhizomes	Rhizomatous initially, becoming stoloniferous with internodes 8-13(-17) cm long	Rhizomes congested, internodes 0.8-1.3 cm long
Stipules	Ovate-triangular, abaxially villous along midrib; margin entire; persist but eventually deciduous	Narrowly triangular, abaxially lanulose-villous; margin ciliate; caducous
Leaf blade		
Size (cm)	14.5-18 × 10.5-14.5	23-38 × 16-32
Adaxial surface	Sparsely reddish-scabridulous and with minute processes	Sparsely setose
Abaxial surface	Tomentose on veins	Lanuginous, particularly pronounced on veins
Inflorescence	Dichasial cymes	Thyrsoid (cymose in weakly developed inflorescence)
Peduncle	Glabrous	Glandular-hispid
Outer tepals	Pinkish; abaxial surface glabrous	Greenish yellow or tinged reddish; abaxial surface sparsely red glandular-hispid
Ovary	Reddish; glabrous	Reddish; red glandular-hispid

**Figure 5:**
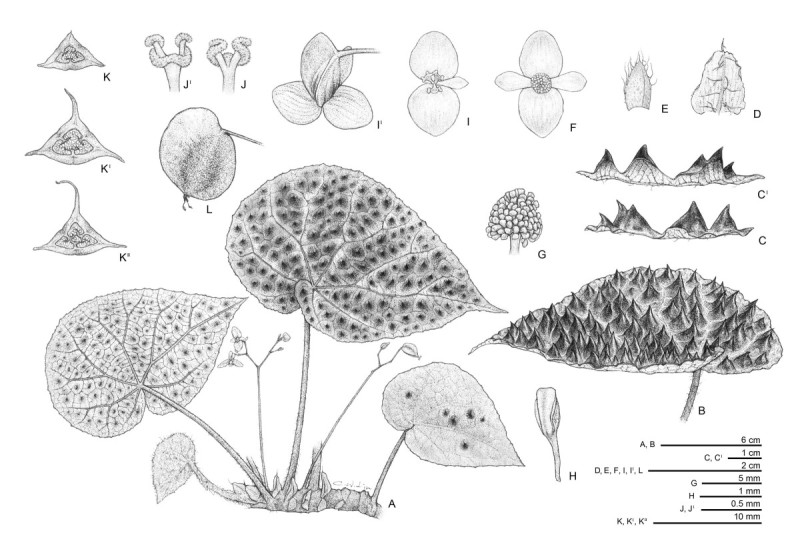
***Begonia ferox*** **C.-I Peng & Yan Liu. A**, Habit; **B**, Leaf; **C**, Leaf cross section; **D**, Stipule; **E**, Bracts; **F**, Staminate flower; **G**, Androecium; **H**, Stamen; **I**, **I’**, Carpellate flower; **J, J’**, Style and stigmas; **K, K’, K”**, Serial cross sections of an immature capsule. All from *C.-I Peng et al. 22956* (HAST).

**Figure 6:**
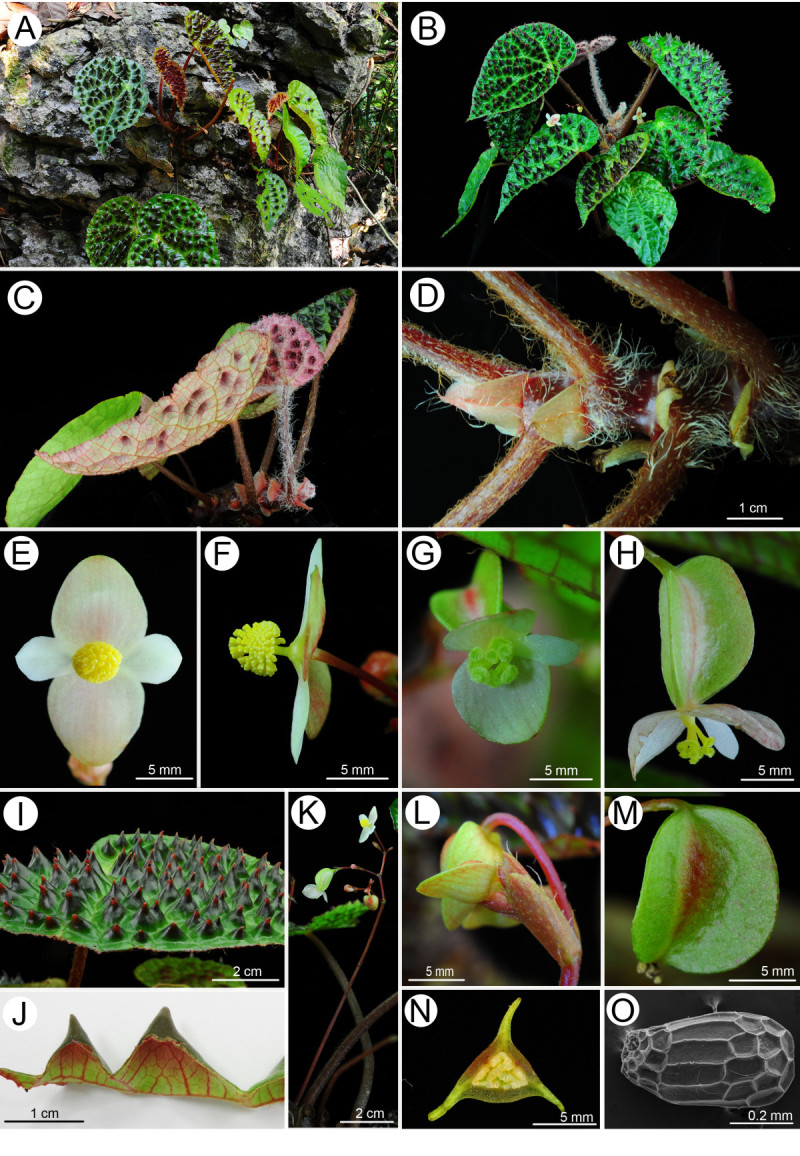
***Begonia ferox*** **C.-I Peng & Yan Liu** ***.*** **A**, Habit and habitat; **B**, Cultivated plant at anthesis; **C**, Leaf abaxial surface and young leaves with dense hairs; **D**, Rhizome and stipules; **E**, Staminate flower, face view; **F**, Staminate flower, side view; **G**, Carpellate flower, face view; **H**, Carpellate flower, side view; **I**, Leaf adaxial surface with bullae; **J**, Leaf cross-section, showing abaxial surfaces of bullae; **K**, Inflorescence; **L**, Bract; **M**, Fruit; **N**, Cross section of ovary; **O**, Seed SEM photomicrograph. All from *C.-I Peng et al. 22956* (HAST).

**2.** ***Begonia ferox*** C.-I Peng & Yan Liu, sp.nov. (sect. *Coelocentrum*) —TYPE: CHINA. Guangxi Zhuangzu Autonomous Region, Longzhou County, Chunxiu Headwater Forest Nature Reserve, elev. ca. 130 m, on forest floor, limestone rock surface, 20 April, 2011. *Ching-I Peng, Kuo-Fang Chung, Yu-Song Huang & Bo Pan 22956* (holotype: HAST; isotypes: E, IBK, PE).  Figures 5 and 6.

**Figure 7:**
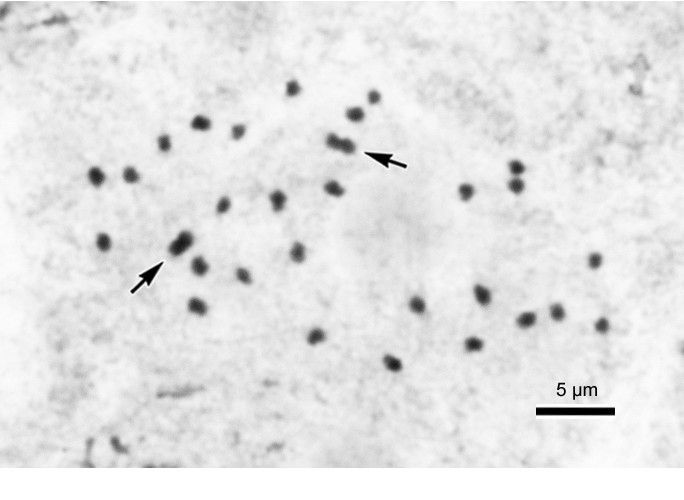
**Somatic chromosomes at metaphase of** ***Begonia ferox*** **C.-I Peng & Yan Liu (2** ***n*** **= 30, from** ***C.-I Peng et al. 22956*** **, HAST).** Arrows indicate a pair of long, metacentric chromosomes.

Monoecious rhizomatous herb. *Rhizome* stout, creeping, 1-2(-2.5) cm thick, to 40 cm long, internodes 1–1.5 cm long, villous near base of petiole. *Stipules* eventually deciduous, ovate-triangular, 1–1.7 cm long, 1.1-1.5 cm wide, herbaceous, strongly keeled, abaxially hairy along midrib, apex aristate, arista ca. 0.2 cm long. *Leaves* alternate, petiole terete, 10-23(-27) cm long, 0.4-0.7 cm thick, villous when young, turning brownish tomentose; leaf blade asymmetric, ovate, (11-)14-19 cm long, 8–13 cm wide, apex acuminate, base strongly oblique-cordate, margin repand, chartaceous, villous when young, adaxially green, surface bullate, intercostal area densely dotted with blackish-brown and hair-tipped bullae, individual bullae conical, tip reddish, (0.3-)0.8-1.3 cm high, (0.3-)0.8-1.2(-1.5) cm across, abaxially pale green, reddish on veins and bullae region, tomentose on veins. *Leaves* of juvenile plant with few or no bullae. *Inflorescences* axillary, dichasial cymes, arising directly from rhizome, branched 3–4 times; peduncle 5–13 cm long, tomentose; bracts and bracteoles caducous, yellowish, bracts narrowly ovate, 1–1.2 cm long, 0.4-0.6 cm wide, boat-shaped, veins reddish, margin fimbriate, bracteoles oblong, ca. 0.3 cm long, 0.1 cm wide. *Staminate flower*: pedicel ca. 1.5 cm long, tepals 4, outer 2 broadly ovate, 0.9-1.1 cm long, 0.6-1 cm wide, abaxially yellowish-reddish, sparsely setulose, inner 2 elliptic, white, 0.7-1.1 cm long, 0.4 cm wide; androecium actinomorphic, spherical, ca. 0.4 cm across; stamens 65–85; filaments fused at base into a column ca. 0.2 cm long; anthers obovate, 2-locular. *Carpellate flower*: pedicel 1.5-1.6 cm long, tepals 3, outer 2 suborbicular or broadly ovate, pinkish-white, 0.8-1.1 cm long, 0.7-1.1 cm wide, inner 1 elliptic, white, 0.8-0.9 cm long, 0.3-0.4 cm wide; ovary trigonous-ellipsoid, 1.3-1.4 cm long, 0.4 cm thick (wings excluded), reddish, 3-winged; wings unequal, greenish-yellow, lateral wings narrower, 0.4-0.5 cm tall, abaxial wing crescent-shaped, ca. 0.6 cm tall, 1.5-1.6 cm wide; styles 3, fused at base, yellow or greenish, ca. 0.4 cm long, stigma spirally twisted. *Capsule* trigonous-ellipsoid, 1–1.5 cm long, 0.2-0.5 cm thick (wings excluded), greenish or reddish when fresh; wings unequal, lateral wings 0.3-0.5 cm tall, abaxial wing crescent-shaped, 0.6-0.9 cm tall. *Seeds* numerous, brown, ellipsoid, ca. 0.5 mm long, 0.3 mm thick. Somatic chromosome number, 2*n*=30 (Figure 7).

#### Chromosome cytology

Somatic chromosomes at metaphase of *Begonia ferox* were determined to be 2*n* = 30 (Figure 7). The chromosome complement of the new species showed a bimodal variation in length. Among the 30 chromosomes, two were longer, ca. 1.8-1.9 μm long (Figure 7: arrows), and the rest of 28 were shorter, ca. 0.7-1.4 μm. The two longest chromosomes were clearly metacentric, however, the centromere positions of most chromosomes could not be determined. Satellites were not observed. All 20 taxa, including *B. longgangensis*, of *Begonia* in sect. *Coelocentrum* that were studied cytologically showed the single chromosome number of 2*n* = 30 (Legro and Doorenbos, 1969; Peng et al., 2012). Among them, eight taxa, namely *B. arachnoidea*, *B. debaoensis*, *B. kui*, *B. ningmingensis* var. *bella*, *B. pengii*, *B. picturata*, *B. umbraculifolia*, and *B. longgangensis* (here reported) have a bimodal variation in chromosome length with two longer metacentric chromosomes. The karyomorphological feature of *B. ferox* reported here agreed with the previous observations.

#### Additional specimens examined

CHINA. Guangxi Zhuangzu Autonomous Region, Longzhou Xian, Chunxiu Headwater Forest Nature Reserve, elev. ca. 130 m, on forest floor, limestone rock surface, 20 April 2011. Flowering specimens pressed from cultivated plants in February, 2013, *Ching-I Peng, Kuo-Fang Chung, Yu-Song Huang & Bo Pan 22956-A* (HAST, IBK).

#### Ecology and distribution

Known only from the type locality in southwestern Guangxi, China (Figure 4). On limestone rocks with abundant leaf litter or on bare rocky slopes in evergreen broadleaf forest, very rare.

#### Phenology

Flowering January-May; fruiting April-July.

#### Etymology

The specific epithet is derived from the fierce-looking leaves with very prominent bullae.

#### Notes

*Begonia ferox* resembles *B. nahangensis* from Vietnam (Averyanov and Nguyen, 2012) in the bullate leaves, differing by the ovate, chartaceous leaves with an acuminate apex; tomentose peduncle not exceeding petioles; and the much larger stature in most vegetative parts. Detailed comparison of the two species is provided in Table 2.

**Table 2:** **Comparison of** ***Begonia ferox*** **with** ***B. nahangensis***

	***Begonia ferox***(Figure5 and6)	***Begonia nahangensis***(based on the protologue of Averyanov & Nguyen,^2012^)
Stipules	1-1.7 cm long, ovate-triangular, strongly keeled with aristae	0.4-0.6 cm long, triangular
Petiole	10-14 cm long, white villous when young, turning brownish tomentose	(2)3-6(10) cm long, densely villous
Leaf blade		
Apex	Acuminate	Obtuse to nearly rounded
Shape	Ovate	Broadly ovate or reniform
Size	11.5-18.5 cm long, 8–12 cm wide	(5)8-12(15) cm wide, usually broader than long
Texture	Chartaceous	Leathery
Inflorescence		
Peduncle	5-10.5 cm long, shorter than petiole, brownish tomentose	8-12(15) cm long, exceeding leaves, glabrous
Carpellate flowers	Outer tepals pinkish-white, suborbicular or broadly ovate, 0.8-1.1 cm long, 0.7-1.1 cm wide; inner 1 elliptic, white, 0.8-0.9 cm long, 0.3-0.4 cm wide	Outer tepals light olive-green, broadly reniform, 0.5-0.6 cm long, 0.9-1.1 cm wide; inner 1 narrowly obovate, 0.5-0.6 cm long, 0.3-0.35 cm wide
Staminate flowers	Outer tepals pale pinkish-yellow, broadly ovate, 0.9-1.1 cm long, 0.6-1.1 cm wide; inner 2 elliptic, white, 0.7-1.1 cm long, 0.4 cm wide	Outer tepals white to light pink (abaxially flushed with brightly red), broadly ovate to almost orbicular, 0.8-0.9 cm long; inner 2 narrowly obovate, 0.4-0.5 cm long, 0.25-0.3 cm wide
Capsule	1-1.5 cm long; abaxial wing crescent-shaped, 0.6-0.9 cm tall	0.8-1 cm long; abaxial wing oblique-triangular, 0.4 cm tall

## Authors’ original submitted files for images

Below are the links to the authors’ original submitted files for images. Authors’ original file for figure 1 Authors’ original file for figure 2 Authors’ original file for figure 3 Authors’ original file for figure 4 Authors’ original file for figure 5 Authors’ original file for figure 6 Authors’ original file for figure 7
