# Supplementary figures and images for: Novelties in Begonia sect. Coelocentrum: B. longgangensis and B. ferox from limestone areas in Guangxi, China
Source: Bot Stud. 2013 Oct 7;54:44. doi: 10.1186/1999-3110-54-44 (PMC5430383; doi:10.1186/1999-3110-54-44)

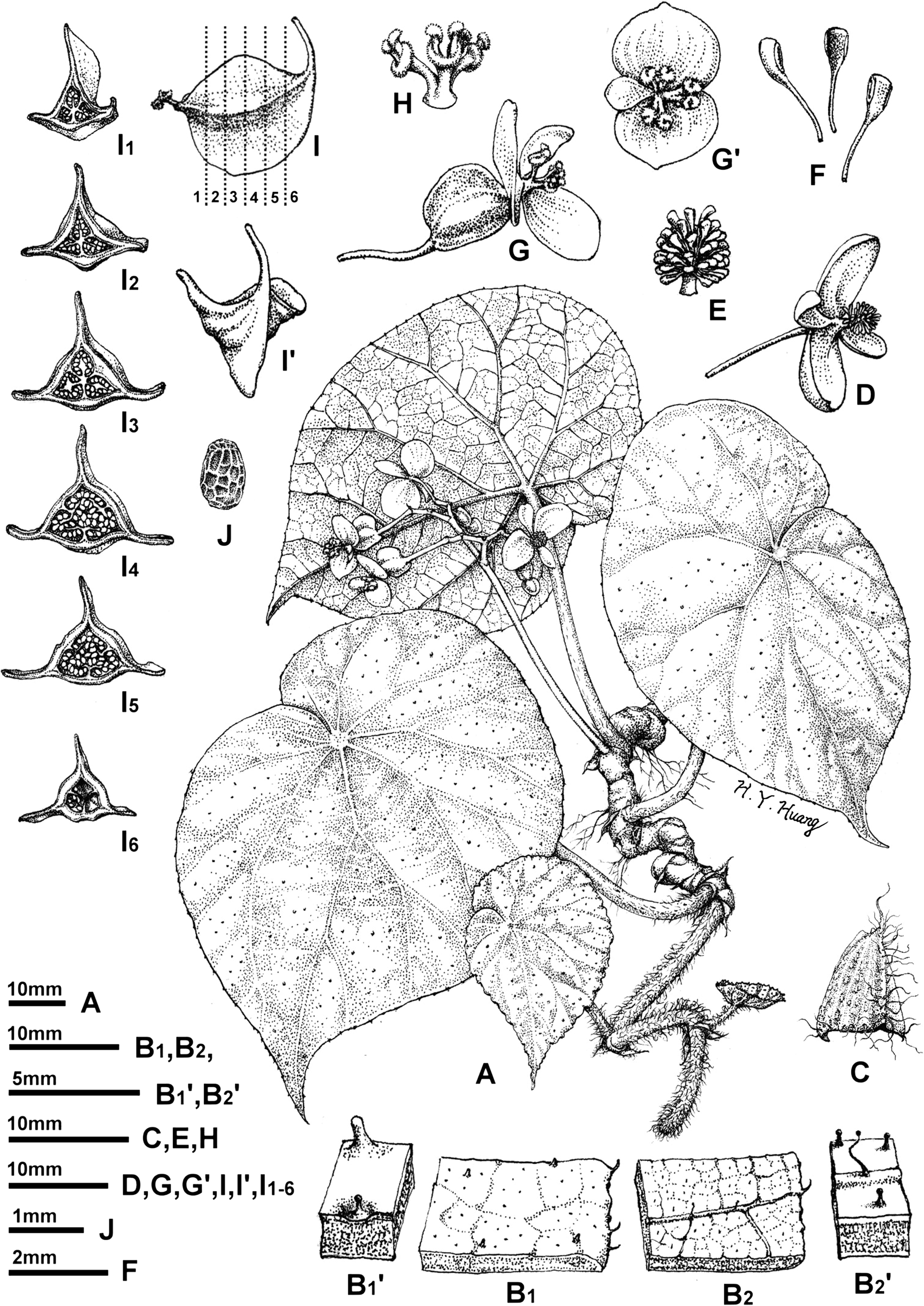

Supplement: Supplementary file 1 — Authors’ original file for figure 1 [file 40529_2013_41_MOESM1_ESM.tif]

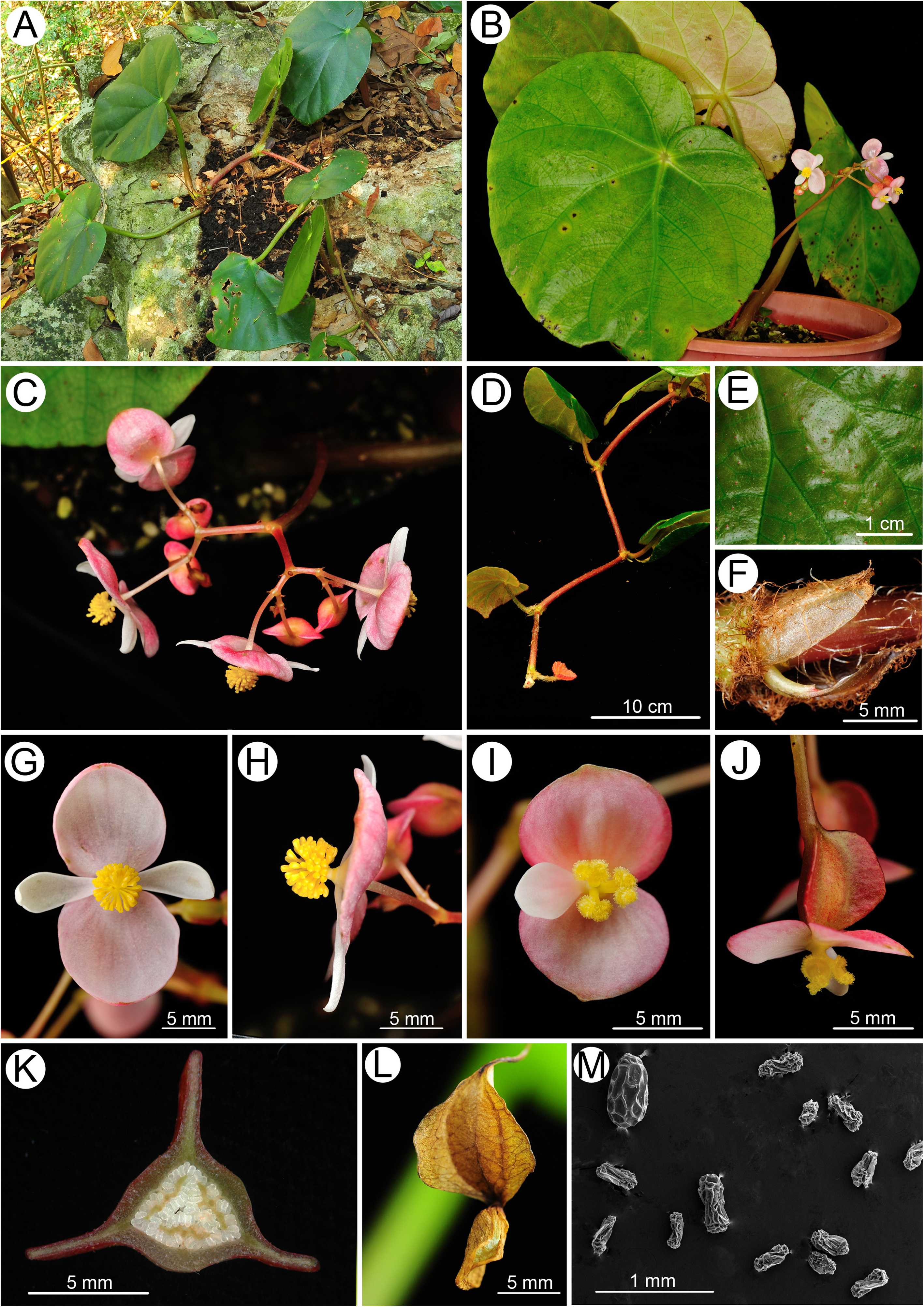

Supplement: Supplementary file 2 — Authors’ original file for figure 2 [file 40529_2013_41_MOESM2_ESM.tif]

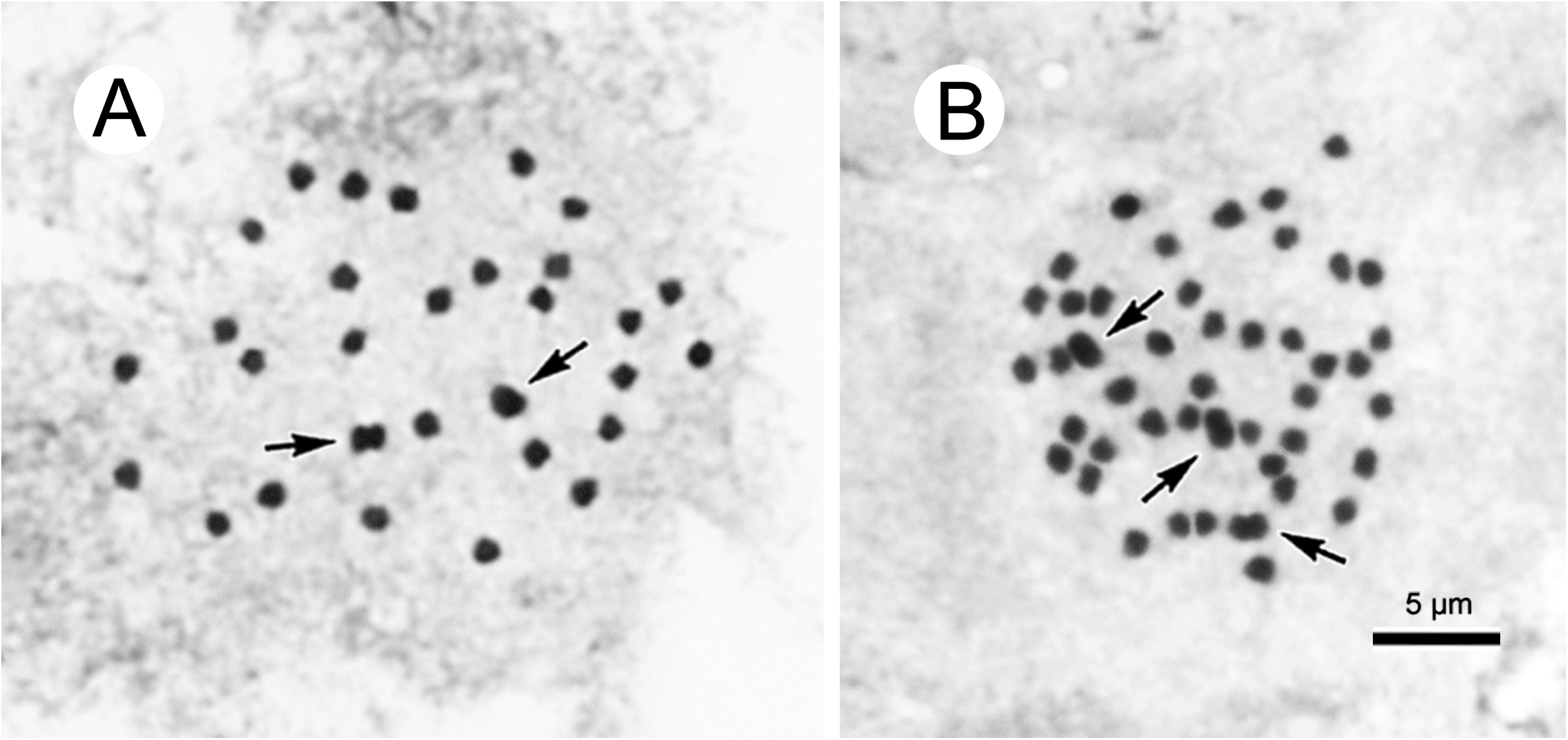

Supplement: Supplementary file 3 — Authors’ original file for figure 3 [file 40529_2013_41_MOESM3_ESM.tif]

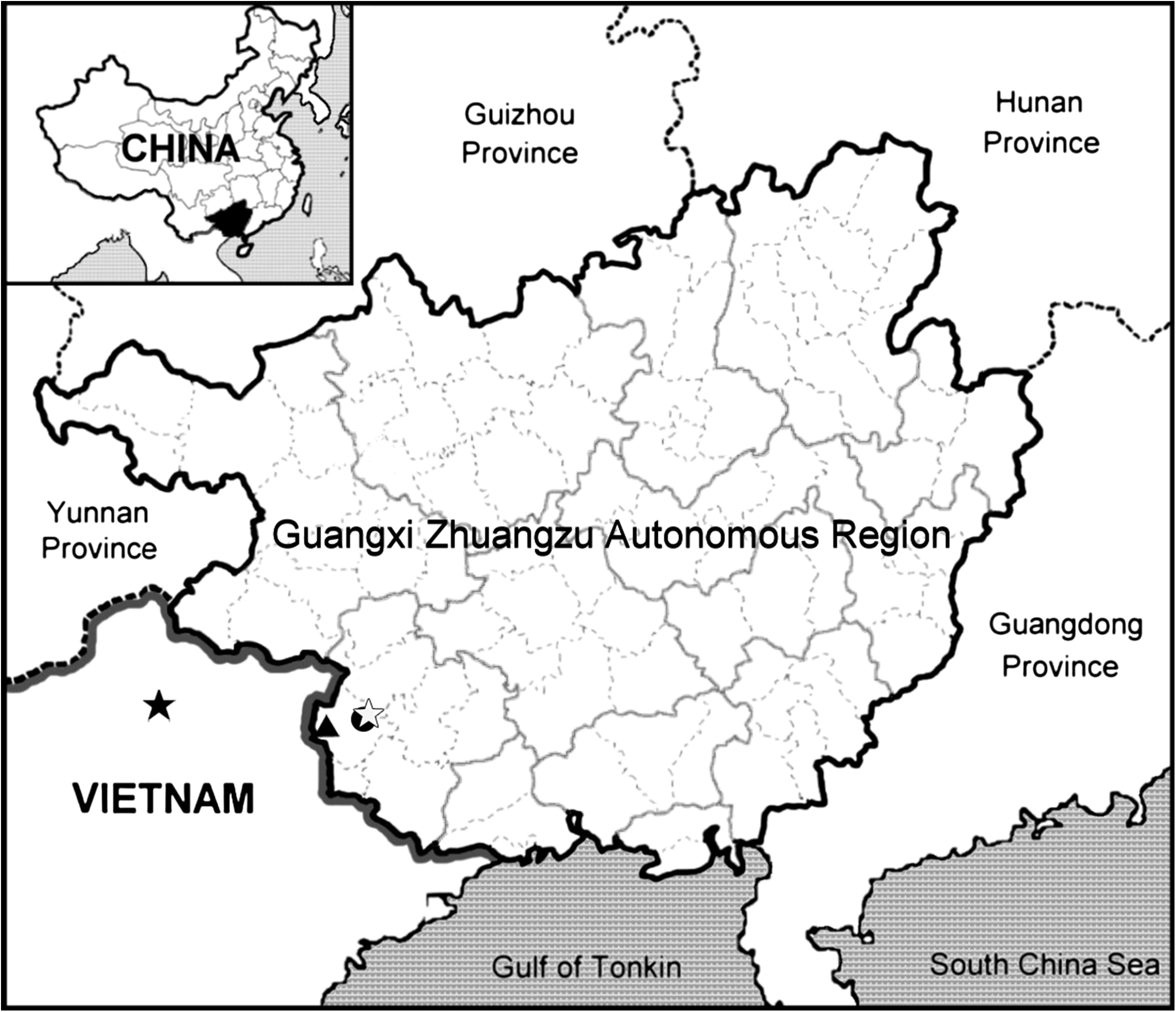

Supplement: Supplementary file 4 — Authors’ original file for figure 4 [file 40529_2013_41_MOESM4_ESM.tif]

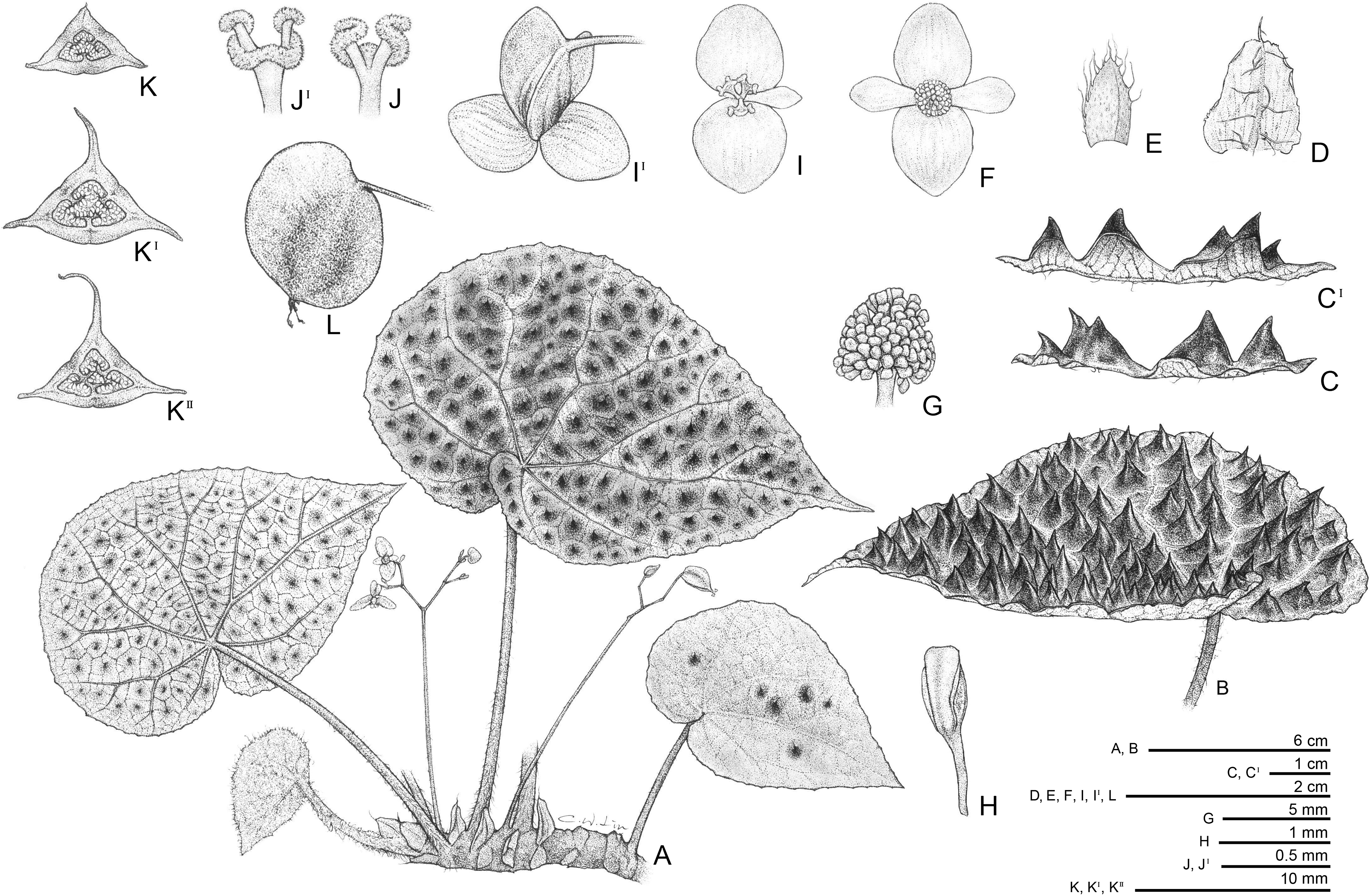

Supplement: Supplementary file 5 — Authors’ original file for figure 5 [file 40529_2013_41_MOESM5_ESM.tif]

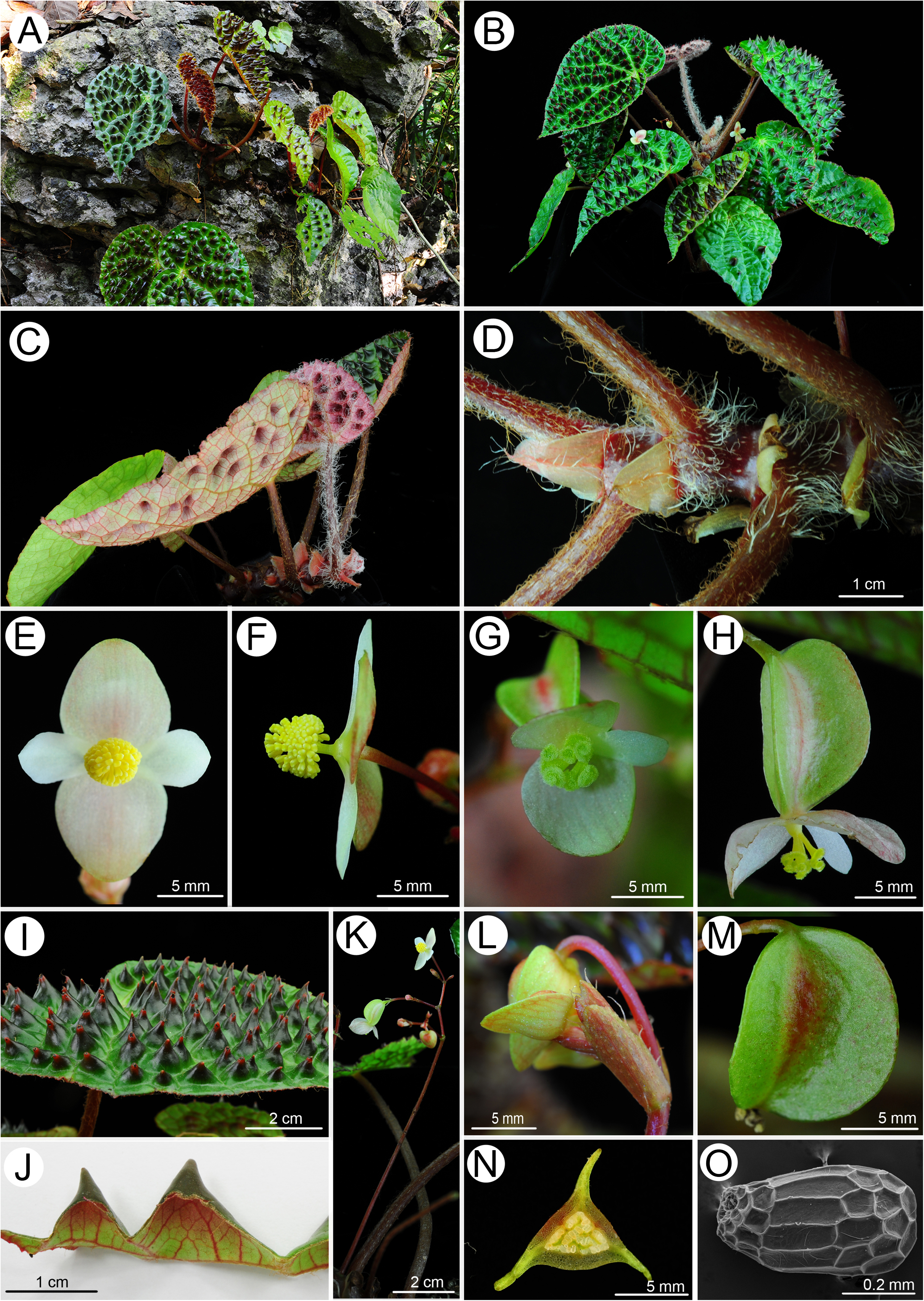

Supplement: Supplementary file 6 — Authors’ original file for figure 6 [file 40529_2013_41_MOESM6_ESM.tif]

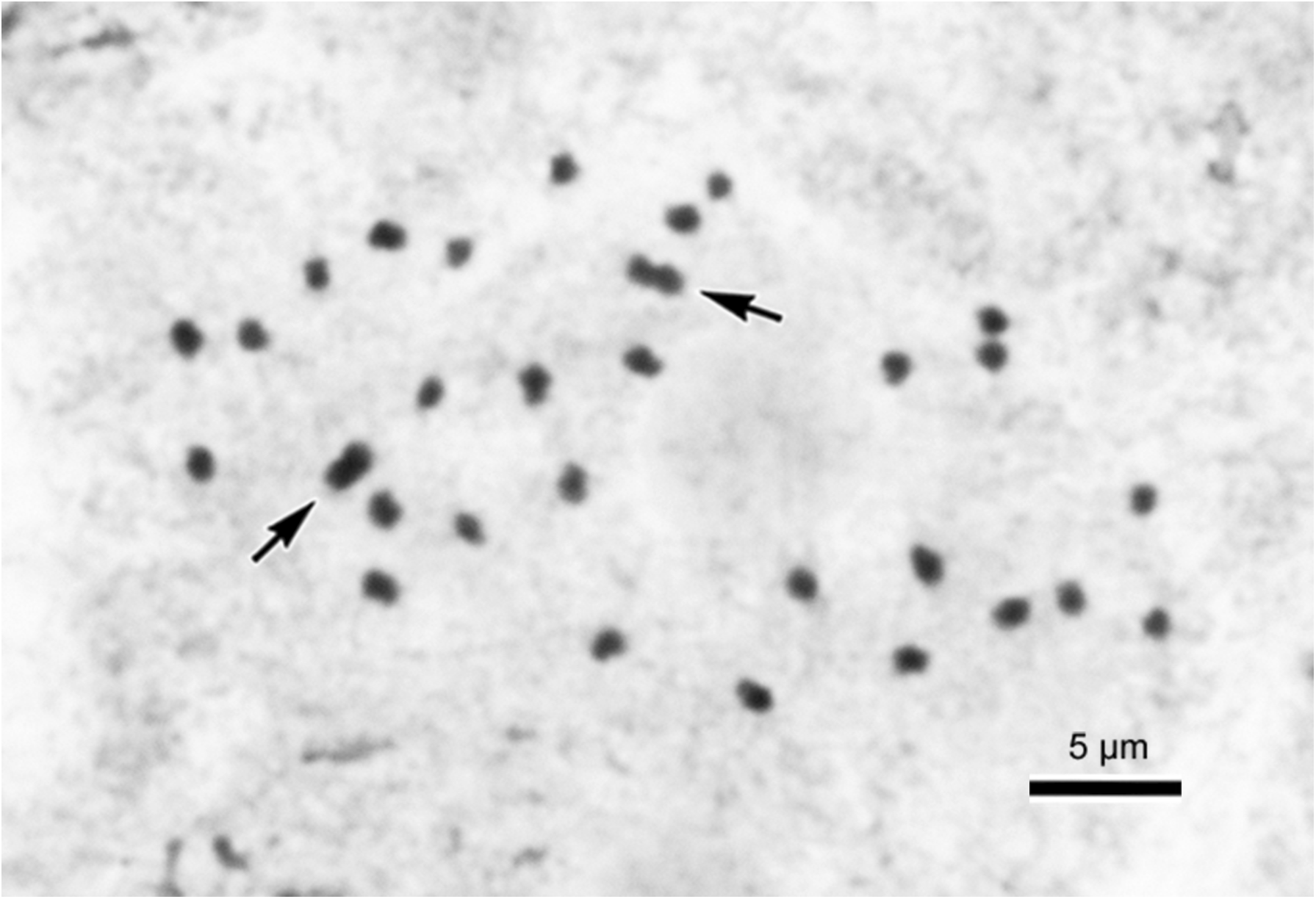

Supplement: Supplementary file 7 — Authors’ original file for figure 7 [file 40529_2013_41_MOESM7_ESM.tif]
